# Palliative care in advanced dementia; A mixed methods approach for the development of a complex intervention

**DOI:** 10.1186/1472-684X-7-8

**Published:** 2008-07-11

**Authors:** Elizabeth L Sampson, Ingela Thuné-Boyle, Riitta Kukkastenvehmas, Louise Jones, Adrian Tookman, Michael King, Martin R Blanchard

**Affiliations:** 1 Marie Curie Palliative Care Research Unit, Department of Mental Health Sciences, Royal Free and University Medical School, London, UK; 2 Department of Mental Health Services for Older People, Barnet Enfield and Haringey Mental Health Trust, London, UK; 3 Department of Mental Health Sciences, Royal Free and University Medical School, London, UK; 4 Department of Palliative Care, Royal Free Hospital NHS Trust, London, UK; 5 Department of Mental Health Care for Older People, Camden and Islington Mental Health and Social Care Trust, London, UK

## Abstract

**Background:**

There is increasing interest in improving the quality of care that patients with advanced dementia receive when they are dying. Our understanding of the palliative care needs of these patients and the natural history of advanced disease is limited. Many people with advanced dementia have unplanned emergency admissions to the acute hospital; this is a critical event: half will die within 6 months. These patients have complex needs but often lack capacity to express their wishes. Often carers are expected to make decisions. Advance care planning discussions are rarely performed, despite potential benefits such more consistent supportive healthcare, a reduction in emergency admissions to the acute hospital and better resolution of carer bereavement.

**Design/Methods:**

We have used the MRC complex interventions framework, a "bottom-up" methodology, to develop an intervention for patients with advanced dementia and their carers aiming to 1) define end of life care needs for both patients and carers, 2) pilot a palliative care intervention and 3) produce a framework for advance care planning for patients.

The results of qualitative phase 1 work, which involved interviews with carers, hospital and primary care staff from a range of disciplines, have been used to identify key barriers and challenges. For the exploratory trial, 40 patients will be recruited to each of the control and intervention groups. The intervention will be delivered by a nurse specialist. We shall investigate and develop methodology for a phase 3 randomised controlled trial. For example we shall explore the feasibility of randomisation, how best to optimise recruitment, decide on appropriate outcomes and obtain data for power calculations. We will evaluate whether the intervention is pragmatic, feasible and deliverable on acute hospital wards and test model fidelity and its acceptability to carers, patients and staff.

**Discussion:**

Results of qualitative phase 1 work suggested that carers and staff were keen to discuss these issues and guided the development of the intervention and choice of outcomes. This will be vital in moving to a phase III trial that is pragmatic and feasible for these complex patients within the NHS

**Trial registration:**

ISRCTN03330837

## Background

Dementia is a progressive neurodegenerative disease which significantly reduces survival [1]. The admission to hospital of a person with dementia with acute medical illness is a critical event: half will die within 6 months [2]. The end of life care received by this group is often poor [3] and patients have inequitable access to palliative care services [4].

Palliative care has been defined as "The active total care of patients whose disease is not responsive to curative treatment. Management of pain, other symptoms and psychological, social and spiritual problems is paramount. The goal of palliative care is achievement of the best quality of life for patients and their families" (WHO). The provision of palliative care services, irrespective of diagnosis, has been supported by a number of recent UK government reports and policies including the 2005 Royal Commission Report [5], the National Audit Office and the End of Life Care Improvement Programme.

Advance care planning discussions are a means by which carers could plan for the future and feel more supported in making decisions regarding patient care. Benefits may include more consistent supportive care, fewer emergency hospital admissions of patients with dementia and better resolution of carer bereavement.

## Design/Methods

### Aims

Our aims are to improve the quality of end of life care received by people with advanced dementia by intervening at the point when patients have an unplanned admission to an acute general hospital.

### Objectives

Our main objective is to assess the feasibility of implementing a palliative care needs assessment and advanced care plan for patients with advanced dementia who have been admitted to the acute medical ward.

We will ascertain which outcome measures are the most valid in this setting. We will also gather information on recruitment, the feasibility of randomisation and attrition in this cohort. We will obtain views from professionals and patients on the acceptability and practicality of delivering or receiving the intervention and monitor the consistency of its functional implementation.

Data will be used to generate the power calculation for a definitive trial and will provide estimates of recruitment rates, barriers to recruitment, the likely treatment effect and reasons for attrition.

### Developing complex interventions

This intervention has been designed using the MRC Complex Interventions Framework [6]. This takes a phased approach involving a series of steps from the pre-clinical research phase (Phase 0) to the final phase IV when the intervention is introduced in the NHS. This leads to theory-driven interventions whose components are well understood. This allows the "bottom up" development of interventions, avoiding hastily embarking on complex and expensive phase III trials that lack the appropriate theory and pilot work.

### Theoretical and pre-clinical work (phase 0)

We used a simple retrospective case note review methodology to demonstrate the poor quality of end of life care that patients with advanced dementia receive when they die on acute medical wards [3]. We then published a systematic review of the literature on palliative care interventions for people with advanced dementia [7]. We found a lack of adequately powered clinical trials in this field and evidence that "palliative care" is often defined as a withdrawal of treatment or of single interventions (i.e. "fever management policies") rather than a holistic approach that formulates a management plan according to individual needs of the patients and their families.

### Modelling phase (phase 1)

#### Preliminary qualitative work

The principal carers of 20 patients with severe dementia, Functional Assessment Staging (FAST) stage 6a and above (difficulty dressing, bathing and using the toilet) [8] admitted to the acute medical ward were recruited and interviewed. Concurrently, 21 health care professionals from a range of disciplines, care settings (acute hospital, nursing home and primary care) and with varying degrees of experience were interviewed. We used framework analysis to identify, extract and analyse core themes [9]. Carers were keen to discuss these issues and it did not cause any undue distress or concerns. In fact, most found the discussion process very helpful.

#### Findings from qualitative work

Five main themes emerged from the data: illness awareness, communication, pain awareness, attitudes towards end of life treatments/quality of life and hospitalisation.

##### Illness awareness

Families were aware that their relative's memory problems would deteriorate but were often unaware of disease progression and the terminal nature of dementia. This lack of understanding was also found in health care professionals, especially junior members of staff. Many did not attribute physical deterioration to dementia.

##### Communication

The provision of information by health care professionals to relatives/carers was mixed. Hospital doctors tended to give information to families regarding the patient's immediate health status while primary care physicians appeared to rely on secondary care to provide relatives with information. A majority of relatives said they wanted more information about the symptoms of advanced dementia, treatment options and planning ahead. A small minority found it distressing to discuss the patient's future. Both professionals and carers were concerned by inadequate communication between primary and secondary care, especially during hospital admission.

##### Pain awareness

Most participants, relatives and healthcare professionals felt confident in recognising pain in patients with dementia although some admitted it was sometimes a "guessing game". Doctors often relied on relatives and nursing staff to guide them. However, the majority of relatives did not think their care recipient was in pain despite many suffering from pressure sores, arthritis and urinary tract infections. Residents in nursing homes commonly had to wait for the GP to assess them which suggests a delay in the provision of adequate pain control. No one mentioned the use of pain assessment tools.

##### Attitudes towards end of life treatments and quality of life

All physicians thought resuscitating patients with advanced dementia was unacceptable. Relatives' views were, however, mixed and mainly depended on religious beliefs, patients' wishes, patients' quality of life or what relatives would want for themselves in similar circumstances. Some felt very worried about the emotional consequences of making such decisions. Attitudes towards feeding tubes also varied between heath care professionals and relatives. Every participant with one exception agreed that patients should always be treated with antibiotics for pneumonia/chest infections.

##### Hospitalisation

Decisions on admitting patients to hospital were influenced by a lack of understanding of dementia as a neurodegenerative illness. Most hospital staff commonly believed that care could be provided equally well in the home or nursing home albeit acknowledging that community services might be reluctant to take responsibility. Nursing home staff felt they must send patients to hospital in response to requests from the GP or the family. Often, relatives favoured hospitalisations initially but later regretted their decision as it became apparent that hospital staff had less time to devote to patient care.

To conclude, a lack of understanding of the natural history of dementia emerged as one major barrier to improving end of life care for patients with advanced dementia. It served to influence attitudes towards treatments and end of life care decisions. This suggests that communication should be at the centre of an intervention attempting to improve end of life care for this patient group, especially providing relatives with adequate information regarding the nature of dementia and its likely progression.

### Exploratory trial (phase 2)

The qualitative data arising from the phase I study have been used to design an intervention for patients and carers that ensures adequate assessment and delivery of palliative care to patients with advanced dementia and includes a framework for discussing advance care planning. This study aims to pilot the intervention in a phase II exploratory/feasibility trial.

#### Study setting

The study is set on acute medical wards for people over the age of 70 years in the Royal Free Hospital NHS Trust, a large inner London teaching hospital serving an inner city population that is diverse in terms of socioeconomic and ethnic mix.

#### Inclusion criteria

These are intentionally broad to ensure that the patients are representative of this population. We will recruit patients with advanced primary degenerative dementia (FAST stage 6a or worse; problems dressing, urinary incontinence and needing assistance with all activities of daily living) who have a high 6-month mortality risk [10]. They will be over 70 years of age with unplanned emergency admissions for treatable acute medical illness. Their carers will be defined as family members or friends who are in regular contact with the patient and who do not act in any professional capacity. They must be next of kin or "key decision makers" and willing to undergo assessment of their own needs and health

#### Exclusion criteria

We will exclude patients without a clearly identified non-statutory carer. Carers should be able to communicate in English to a degree whereby they can participate in the interviews and assessments (unfortunately limited funding precludes the provision of interpreters). We will also need to exclude carers who do not have full mental capacity to give consent to participate in research.

#### Patient recruitment

The research team will screen all admissions to two acute wards (intervention and control) and identify potential participants. We will monitor recruitment rates per month in relation to the total numbers of patient/carer dyads who would be eligible and detail reasons for those who decline to participate. We intend to recruit 40 patients and carers to the intervention arm and 40 patients and carers to the control arm. For an overview of measurements taken during the study, see Table 1.

**Table 1:** Study schedule

**Measure**	**Source**	**Baseline**	**6 weeks**	**6 months**	**Subsequent hospital admission**	**Time of death**	**3 months post bereavement**
**Carer related**							

KD-10	Carer	**x**	**x**	**x**			**x**
Satisfaction with end of life care in dementia scale	Carer						**x**
Decision satisfaction inventory	Carer	**x**	**x**				**x**
EQ 5D	Carer	**x**	**x**	**x**			**x**
Visual analogue scale	Carer	**x**	**x**	**x**			**x**
Economic data	Carer	**x**		**x**			

**Patient related**							

Pain scale measurements	Patient						
Painful interventions scale	Patient care records	**x**			**x**	**x**	
Other interventions	Patient care records	**x**			**x**	**x**	
Quality of end of life care	Patient care records	**x**				**x**	
Adherence to LCP	Patient care records	**x**				**x**	

**System related**							

Numbers choosing to make ACP	Patient care records	**x**				**x**	
Adherence to advance care plan	Patient care records					**x**	
Referrals to palliative care team	Patient care records	**x**			**x**	**x**	
Unplanned admissions	Patient care records			**x**	**x**	**x**	
Place of death	Patient care records					**x**	
Economic data	Patient care records	**x**		**x**		**x**	

### Intervention

#### Feedback and refinement of pilot intervention

Because the intervention is based on the fundamental principles of palliative care, it will remain relatively constant. We will, however, test alternative *formats *of the intervention in order to obtain the most pragmatic and clear version. The intervention will be piloted on 5 patients and their carers. A chartered health psychologist, employed for the whole study, collected the phase 1 qualitative data. She will obtain informal feedback from carers and key staff in the medical multi-disciplinary team (MDT). The next version will be piloted on a further 5 patients and their carers and modified in accordance with findings until a final format is reached.

During the pilot trial, we shall seek practitioners' and carers' views of the intervention by using semi-structured interviews. These data will be used iteratively to optimise implementation of the intervention. We shall assess whether carers and professionals regard the intervention as coherent, understandable, acceptable and potentially integral to the service. We shall also examine change management and the factors that make implementation difficult in each setting [11]. If necessary, we will seek permission from the ethics committee to make substantial amendments to the protocol.

#### Study procedures-Control arm

The patients and carers in the control arm will be recruited from an adjacent ward. They will be fully informed about the trial but there will be no change to usual care. Identical outcome measures will be used for both control and intervention groups. If the trial reveals areas of clinical need the MDT will be informed and this intervention documented.

#### Study procedures-Intervention arm

The intervention will be delivered by a clinical research nurse experienced in the care of patients with dementia and acute medical illness and dealing with discussions of a sensitive nature who has received training in specialist palliative care. The nurse will assess the patients and carry out the discussion and advanced care planning with the carers. She will liaise with the clinical team and others involved in the patients' care, for example the GP or nursing home. In addition patients and carers in this arm will receive standard hospital treatment.

#### Stage 1: Assessment of patient

Most elements of the patient assessment could be considered routine clinical care. However, we aim to formalise and integrate this information into a more specialised evaluation. The assessment will also inform the discussion with the carer. The holistic assessment will take approximately 30 minutes and will cover a range of domains including dementia severity, the presence of delirium, communication, pressure sores and skin condition, food and fluid intake, swallowing and feeding (see Table 2).

**Table 2:** Patient assessment

**Dementia Severity** *Functional Assessment Staging (FAST) Score *[8]	An observational scale describing a continuum of 7 successive stages from normal to the most severe dementia
**Delirium** *Confusion Assessment Measure (CAM) *[18]	The most widely used instrument for the detection of delirium. It has a sensitivity of 94–100% and a specificity of 90–95%.
**Pain and discomfort**	
*Abbey Pain Scale *[19].	A brief 6-item scale to measure the intensity/severity of acute and chronic pain in late stage dementia
Pain Assessment Checklist for Seniors with Severe Dementia (*PACSLAC*) [20]	Measures the number of pain symptoms present
*Doloplus-2 *[21]	A 10-item observational scale for use in non-verbal adults. Addresses a wide range of pain indicators
**Communication**	Clinical assessment of patient's ability to communicate their needs including non-verbal communication and comprehension.
**Pressure sores and skin condition** *Waterlow Scale *[22] *Stirling Scale *[23]	The Waterlow scale is routinely used for the assessment of risk for developing pressure sores. The Stirling Scale measures the extent of damage from 1, Non-blanching erythema of intact skin to 4, full-thickness wound, which involving subcutaneous tissue and the deep fascia.
**Mobility**	Is the patient bed bound/able to turn themselves? Can they walk with/without use of aids i.e. Zimmer frame?
**Elimination**	Continence is routinely assessed as part of the FAST scale. Patients will also be assessed for the presence of urinary tract infection and constipation.
**Food and fluid intake**	As routinely documented on food and fluid chart
**Swallowing and feeding**	Formal assessment by speech and language therapy will be requested if there is clinical suspicion of difficulty.

The measurement of pain in people with dementia is difficult. We aim to measure both the severity and number of pain symptoms present. There are no dementia pain scales validated for use in an acute hospital setting. We will, as part of this pilot study, evaluate a range of pain scales in acutely unwell patients with dementia. The scales are simple and require only concurrent observation of the patient or the opinion of current caregivers and staff.

This will generate a list of the patient's active problems that will be discussed with the clinical team. A management plan will be formulated and will be documented in the clinical notes. If patients are identified as being in the terminal phase (last 48 hours of life), this will be discussed urgently with the clinical team who may decide that the patient should be transferred to Liverpool Care Pathway [12].

#### Stage 2: Assessment and discussion with carer

This will take the form of up to two structured consultations (at least 5 days apart) and based on principles underpinning palliative care (physical, social, psychological, spiritual, cultural, information) and on the findings of the qualitative phase. This discussion will be initiated on the acute medical ward but if the patient is discharged will be completed in the community.

In the first consultation, we will obtain a baseline understanding of the current situation and context of both patient and carer and carry out an assessment of needs. We will assess the carer's level of knowledge about the patient's dementia, level of severity and prognosis, and ask them to identify the patient's **physical **needs of most concern to them.

The **social **situation will be evaluated in terms of family dynamics and defining the key decision makers regarding the person's care, the current level of support, date of most recent needs assessment from social services, and carer's unmet health, social and financial needs. We will assess the level of **psychological **support that the carer has available to them and **spiritual **and cultural factors such as the faith background of both patient and carer. We will assess the **information **available such as whether the person with dementia made any previous advanced directives or expressed any opinions in the past, for example, preferred place of care at time of death.

The discussion will be concluded by summarising and acknowledging the carer's role and the main issues discussed. This will be documented using a standardised form. Any issues of serious concern will be discussed with the medical multidisciplinary team and this intervention documented.

In the second consultation we will provide and discuss information. This will take account of, and respect, the patient and carer's individual circumstances established in the first interview. We shall start by giving basic education on dementia as a neurodegenerative disease and provide carers with information on the prognosis of advanced dementia, palliative care (focusing on palliative care as appropriate active care and management, NOT withdrawal of treatment) and advanced care planning. Carers will then be given the opportunity to make an advanced care plan for the patient if they wish to do so.

The advanced care planning phase of the intervention was developed using the phase I qualitative work and a number of current UK Department of Health guidelines. Advance Care Planning (ACP) has been defined as a process of discussion between an individual and their care providers. This includes important values or personal goals for care, understanding about illness and prognosis and preferences for types of care or treatment that may be beneficial in the future and the availability of these [13]. We will pilot an adapted version of a tool developed by the UK National Health Service (Preferred Priorities for Care) [14]. We will use this tool to address specific issues identified by the patient assessment and the initial carer discussions.

Care planning involves the discussion of sensitive and potentially upsetting issues and carers may require ongoing support and advocacy for further decision making. Whether or not the carer decides to make an advance care plan, the nurse will end the process by signposting or facilitating referral to services so that unmet patient and carer needs can be addressed. Carers will be given a contact card for the research nurse. The advanced care plan with statement of preference and wishes will be written up and if the carer consents, copies will be placed in the medical notes, sent to the GP (and if relevant the nursing home) and kept by the carer.

### Outcome measures

This is a pilot study, so we will examine the feasibility of a number of outcome measures to identify those with most utility for a future phase III randomised controlled trial. Four main domains of outcomes will be assessed (Table 3).

**Table 3:** Study outcome measures

**Carer**	
**Stress and wellbeing** *Kessler Distress Scale *(*KD10*) [24]	10-item scale, validated and widely used screening tool for psychological distress. It is discriminative in detecting "caseness" for DSM-IV disorders
**Health Status** *(EQ-5D) *[25].	Standardised, short, 5-item scale instrument for health and quality of life
**Decision-making process** *Decision Satisfaction Inventory *[26]	Validated measure with three sub-scales measuring overall satisfaction with medical care, satisfaction with the decision making process, and satisfaction with the decisions made
**Satisfaction with decision-making process-***Decision Conflicts Scale*	Validated, 16-item scale measures uncertainly and difficulties in the decision making process [27].
**Satisfaction with care** *Satisfaction with End of Life Care in Advanced Dementia Scale *[28]	Specifically developed for use in advanced/end stage dementia. Three separate domains of measurement; satisfaction with the terminal care, symptom management, comfort during the last 7 days of life. Only to be completed after the patient has died.
**Visual analogue scales**	A standard 10 cm visual analogue scale to measure carers satisfaction with: 1) the process of the advanced care planning 2) the utility/usefulness of the advance care planning
	
**Patient**	
**Active interventions** *Painful Interventions Scale *[29]	Measures invasive hospital procedures shown to be moderately to severely painful or uncomfortable including arterial blood gas testing, mechanical ventilation, naso-gastric feeding and bladder catheterisation
**Other interventions**	i.e. Resuscitation status, PEG feeding, prescription of neuroleptics
**Quality of end of life care**	Prescription of analgesia at time of death, use of Liverpool Care Pathway
**Survival times**	Time of intervention to time of death
**System**	
**Advanced care planning**	Numbers choosing to make advanced care plan and adherence to advance care plan
**Readmission rates**	For emergency acute admissions
**Place of death**	
**Referrals**	To community palliative care
**Economic**	
**Service use costs **[30] Client Service Receipt Inventory (CSRI)	Baseline, 6 months and, if relevant, for the month prior to death

1) *Carer related *– such as stress and well being, general health and quality of life, satisfaction with the decision making process and a general measure of how they experienced the care planning process.

2) *Patient related *– to include measures of painful medical interventions i.e. intravenous cannulation, measurement of arterial blood gases), indicators of the quality of end of life care and survival time (intervention until death). These do not involve any burden to the patient and are gathered from routine clinical documentation.

3) *System-related *– for example adherence to the advanced care plan, referrals to and input from community palliative care, rates of unplanned hospital admission, and place of death.

4) *Economic measures *– differences between intervention and control groups may have an economic impact (positive or negative) in terms of health/social care resources used and also help provided by informal carers. Data on hospital stays will be obtained from administrative records.

#### Follow-up

The timing of patient and carer follow up assessments has been determined by the published evidence on survival time and prognosis for these patients (see Table 1). We will assess the feasibility of long term follow up i.e. whether reliable data can be collected about the care received at death. We will follow up patients and carers at 6 weeks from the index admission, at any subsequent hospital admissions and at 6 months from baseline. We aim to interview carers again three months after bereavement.

#### Data management and analysis

We shall use descriptive statistics to examine baseline data on proportions of carers agreeing to assessment and patients' demographic and clinical characteristics. Follow-up rates will be reported. Outcome measures, including clinical and health economic outcomes will be explored using descriptive statistics. Differences between the intervention and control groups will be investigated using regression methods to control for baseline characteristics. We will estimate the likely effect size of the intervention (additional to usual care) in order to guide a sample size calculation for a definitive phase III randomised trial. Economic factors will also be evaluated; unit costs [15] will be estimated for each of the study arms and the cost for each patient calculated. These will be linked to the short-term outcomes in the form of an incremental cost-effectiveness analysis that will enable us to make preliminary comparisons between the intervention and control groups in terms of the cost of achieving unit changes in outcomes. Cost comparisons between the two arms will be made using bootstrapping methods if data are skewed.

#### Ethical issues

By definition, this research will be carried out on subjects who are unable to give informed consent. Our study was approved by the ethics committee of Camden and Islington Mental Health and Social Care Trust (London, UK). This ethics committee is "flagged" to consider research on those unable to consent for themselves, as described in Section 30 of the Mental Capacity Act 2005. We believe that the risk to patients and carers is negligible and this project does not involve any invasive procedures. We will consult and seek assent from the carers and any other relevant people as defined by section 32 of the Mental Capacity Act (2005) code of practice. As we will be measuring the impact of the intervention on specific carer outcomes we will seek fully informed consent from the carer for their own participation as a subject.

## Discussion

Patients with advanced dementia have complex physical and psychological needs. They are different to many other terminally ill patients in that they have severe impairment of basic activities of daily living and may lack capacity to make appropriate decisions regarding their choices and preferences at the end of life. Assessing needs at the end of life and planning care is a complex issue and, in people with advanced dementia, much of this may be done by proxies. These difficulties are further increased by the healthcare system. People with advanced dementia may reside in nursing homes and at the same time be under the care of acute hospitals, community mental health teams and clinical staff from a range of specialities spanning primary and secondary care. This complicated care system makes adequate communication between care providers, patients and their carers very challenging.

We have used the MRC complex interventions framework to move from our earlier theoretical work to pilot an intervention that aims to address the issues described above. The findings from the qualitative phase were vital in investigating and teasing out the important "ingredients" of an intervention. It highlighted challenges to improving end of life care for this patient group. In particular, it demonstrated a widespread lack of understanding of the natural history of dementia and its progression. This suggests that education and advanced care planning discussions should be at the centre of an intervention. Benefits may extend to improved understanding, more appropriate decision making and thereby reductions in un-necessary hospitalisations as well as more appropriate end of life care for patients with advanced dementia. It may also lead to greater satisfaction with end of life care and better bereavement outcomes in relatives.

We shall use this pilot phase to evaluate whether the intervention is pragmatic and feasible within the challenging environment of NHS acute hospital wards. It will be enable us to determine which are the most effective and useful outcome measures and what may be the best measures of effectiveness; these may differ between the carer, potential benefit for the person with dementia and wider benefit to the health service. We do not know which aspect of the intervention will provide the active component that most influences change within this complex system. We envisage that to avoid "contamination" the future phase III trial will be clustered at hospital ward level. By defining key primary outcomes we will be able to generate data for a power calculation, which is vital as we anticipate that cluster randomisation will be the optimal methodology.

The preparatory work allowed us to explore ethical issues regarding research on subjects who do not have the capacity to give fully informed consent to participate. We used an ethics committee "flagged" to deal with research on subjects who lack the capacity to consent (under the terms of the recently enacted Mental Capacity Act) and will gain assent from their carers. We believe that this research brings a number of potential benefits as determined by the Act, for example "developing more effective ways of treating a person or managing their condition", "improving the quality of healthcare, social care or other services that they have access to" and "reducing the risk of the person being harmed, excluded or disadvantaged".

There are a number of methodological issues that still require consideration. We do not know what constitutes a good outcome in these patients with advanced dementia who are generally unable to express their needs and wishes. We will use the views of proxies which may not accurately reflect patients' wishes when they last had capacity.

It could be argued that much of what comprises this intervention is just good quality care and should be part of routine clinical practice; it is too much like what professionals believe they "do already". Naturally, some elements of the intervention will occur in usual care, although all the evidence published to date suggests that it is rarely carried out in a consistent manner. Furthermore, this sort of concern hardly ever interferes with a careful, controlled evaluation [16]. In addition to evaluating the intervention itself, we will also assess its implementation in terms of change management within a complex health [17] system.

The results of our study may have wider implications, particularly with regards to palliative care interventions in neurodegenerative disease and conditions other than cancer where patients may lack capacity and the disease trajectory is not easy to predict.

## Competing interests

The authors declare that they have no competing interests.

## Authors' contributions

MRB, ELS, MK, AT and LJ obtained funding for the project. MRB, ELS, IT-B, RK, MK designed the intervention. IT-B carried out the phase 1 qualitative work. RK and IT-B are implementing the phase II trial. ELS drafted the paper and all authors read and approved the final manuscript.

## Pre-publication history

The pre-publication history for this paper can be accessed here:


